# Isolation, Identification, and Genetic Phylogenetic Analysis of Two Different Genotypes of Bovine Parainfluenza 3 Virus in China

**DOI:** 10.3390/v14102221

**Published:** 2022-10-09

**Authors:** Xu Wang, Jianjun Hu, Fanyan Meng, Yiheng Cao, Zijie Wang, Qianyi Zhang, Qian Zhang, Xingxing Zhang, Mengli Han, Tongzhong Wu, Fagang Zhong, Xin Huang

**Affiliations:** 1 Xinjiang Academy of Agricultural and Reclamation Sciences, State Key Laboratory of Genetic Improvement and Healthy Breeding of Sheep, Shihezi 832000, China; 2 College of Animal Science and Technology, Tarim University, Aral 843300, China; 3 China Animal Husbandry Industry Co., Ltd., Beijing 100070, China; 4 China Veterinary Drug Control Institute, Beijing 100081, China

**Keywords:** bovine parainfluenza virus type 3, genetic phylogenetic analysis, virus identification, indirect immunofluorescence, virus isolation, next-generation sequencing

## Abstract

Bovine parainfluenza virus 3 (BPIV3) is one of several viruses that contribute to bovine respiratory disease complex (BRDC). During this study, isolation of BPIV3 was attempted from 20 PCR-positive swabs by Madin-Darby Bovine Kidney (MDBK) cells. Nine samples showed obvious cytopathic lesions identified as BPIV3 by reverse-transcription polymerase chain reaction amplification and sequencing. The genomes of isolates XJ21032-1 and XJ20055-3 were sequenced using Illumina sequencing technology and determined to have lengths of 15,512 bp and 15,479 bp, respectively. Phylogenetic analysis revealed that isolate XJ21032-1 was genotype B, and isolate XJ20055-3 was genotype C. In addition, the two isolates had multiple amino acid changes in nucleocapsid protein, fusion protein, and hemagglutinin/neuraminidase, major antigenic proteins. This allows the further recognition of the presence of BPIV3 type B in Chinese cattle herds. We hope this will help trace the origin of BPIV3, improve the understanding of differences between genotypes, and provide data support for vaccine development.

## 1. Introduction

Bovine parainfluenza virus 3 (BPIV3) is one of several viruses that contribute to bovine respiratory disease complex (BRDC). Under stressful conditions, cattle are highly susceptible to BPIV3 infection, which weakens their immunity and leads to secondary bacterial and mycoplasma infections, eventually causing BRDC [1]. Additionally, it has been reported that BPIV3 shows cross-species transmission, including humans [2] and sheep [3]. BPIV3 was first reported in the United States in 1959 [4], where it was first isolated. Since then, it has been reported in many countries worldwide. In Canada, Peter et al. [5] tested 1745 serum samples from 295 farms for BPIV3 antibodies using an enzyme-linked immunosorbent assay and found a positivity rate of 93.3%. In Turkey, Muftuoglu et al. [6] tested 1307 serum samples randomly collected from 2015 to 2019 using a standard virus neutralization test and found a positivity rate of 56.2%. Newcomer et al. [7] performed neutralization tests of three BPIV3 genotypes using sera from five unimmunized herds in the United States. They found that antibody titers were significantly higher for genotype B than for genotypes A and C. In China, Wang et al. [8] tested 2489 bovine serum samples from 12 provinces for BPIV3 antibodies and found a positivity rate of 77%, indicating that BPIV3 is widespread in China.

BPIV3 belongs to paramyxoviridae, the genus respirovirus. These are non-segmented, single-stranded, negative-strand RNA viruses with genomes encoding the following six structural proteins: nucleocapsid protein (N); phosphoprotein (P); matrix protein (M); fusion protein (F); hemagglutinin/neuraminidase (HN); and large polymerase protein (L). The BPIV3 genome sequence is currently classified into three genotypes, A, B, and C, based on phylogenetic analyses [4,9,10]. Type A was first discovered in the United States [4] and later in China [11,12], Japan [13], Egypt [14], Argentina [15], and other countries. Type B was first discovered in Australia [9] and later in cattle in the United States [16] and buffalo herds in Argentina [15]. Type C was first found in China [10] and was later isolated in the United States [16], Korea [17], Japan [18], Turkey, and other countries [19]. The discovery of different genotypes of BPIV3 in multiple countries indicates that it has spread worldwide.

Although some studies of BPIV3 have been performed in China, there has been little information about BPIV3 surveillance in the Xinjiang province. In the present study, nine BPIV3 isolates were identified. Among them were XJ21032-1 and XJ20055-3, which were identified by immunofluorescence assay and morphology. The genomes were sequenced using next-generation sequencing technology. Phylogenetic analysis showed that the XJ21032-1 and XJ20055-3 isolates were genotypes B and C, respectively. When XJ21032-1 was compared with the type B reference strain Q5592 (EU277658), and when XJ20055-3 was compared with the type C reference strain NX49 (KT071671), we observed a variety of amino acid changes in the main antigenic proteins N, F, and HN. This is the first report of the type B strain of BPIV3 in China. This study aimed to elucidate BPIV3 infection and its prevalent subtypes in calf herds at cattle farms in Xinjiang and provide a theoretical basis for the tracing, prevention, and control of BPIV3 in Xinjiang.

## 2. Materials and Methods

### 2.1. Sample Collection

To implement the calf health service plan in Xinjiang, we cooperated with 14 cattle farms to investigate the pathogen in calves. Four hundred seven nasal swabs from Holstein calves (0–90 d old) were randomly collected by M.H., F.Z., and X.H., veterinarians with at least ten years of experience, in seven regions (Shihezi, Tacheng, Urumqi, Karamay, Bazhou, Aksu and Ili) in the Xinjiang Uygur Autonomous Region, China, from 2020 to 2021. Approximately 5–20% of the calf population on these farms showed clinical signs of mild pneumonia during sample collection.

### 2.2. Polymerase Chain Reaction Assay

Oligonucleotide primers for infectious bovine rhinotracheitis virus (IBRV), bovine viral diarrhea virus (BVDV), bovine respiratory syncytial virus (BRSV), and BPIV3 detection and identification are shown in Table 1.

Total RNA was extracted from nasal swabs or cell culture fluids of virus isolates using TaKaRa MiniBEST Viral RNA/DNA Extraction Kit Ver.5.0 (TaKaRa, Shiga, Japan), according to the manufacturer’s instructions. The reverse transcription was carried out in the PrimeScript™ RT reagent kit (TaKaRa, Shiga, Japan) according to the instructions using 4 μL of RNA sample and RT Primer Mix as a reverse transcription primer.

The amplification of cDNA by PCR was carried out in a total volume of 25 μL containing 2× ES Taq MasterMix (CWBIO). The reaction was heated in a thermocycle for 5 min at 94 °C and then submitted to 35 cycles of 1 min at 94 °C, 1 min at the required temperature for each primer pair (Table 1), 45 s at 72 °C, and a final 10 min incubation at 72 °C. The amplification products were sequenced by Anhui General Co. (Chuzhou, China).

### 2.3. Virus Isolation

Each nasal swab was soaked in 1 mL of phosphate-buffered saline (PBS), and the resultant fluid was centrifuged at 4000 rpm for 10 min. It was then filtered through a 0.22-µm filter. The filtrate was inoculated into MDBK cells (MEM, GIBCO) that had been well-grown to 80% confluence and incubated for 2 h at 37 °C under 5% CO_2_, with gentle shaking at 30 min intervals. Then, the culture solution was discarded, 2 mL of PBS were added, and the 25 cm^2^ culture flask was gently shaken to ensure complete contact between PBS and the cells adsorbed on the bottom of the flask. Next, the washing solution was discarded. The flask was washed three times. Then, 5 mL of serum-free DMEM (Sangon Biotech) were added and incubated at 37 °C under 5% CO_2_. The cells were observed every 12 h. If cytopathic effects (CPEs) were observed, the flask was removed from the incubator when 70% of the cells had developed CPEs. It was then freeze-thawed three times using a −80 °C refrigerator. Then, the culture was collected in a centrifuge tube and centrifuged at 6000 rpm for 10 min. The supernatant was collected and stored in a −80 °C refrigerator. If no CPEs were generated, the culture was passaged. The sample was classified as negative if no CPEs were observed after three consecutive passages.

### 2.4. Indirect Immunofluorescence Test

One strain was selected from two groups of different genotypes, XJ21032-1 and XJ20055-3. The solutions containing the XJ21032-1 and XJ20055-3 isolates were inoculated into monolayers of MDBK cells well in confocal culture dishes and incubated for 2 h at 37 °C under 5% CO_2_ with gentle shaking at 30 min intervals. The viral solution was discarded after adsorption, 2 mL of PBS was added, and the dish was gently shaken to ensure complete contact between the PBS and the cells adsorbed at the bottom of the dish. The washing solution was discarded, and the dish was washed three times. Next, the PBS was discarded, and 500 μL of serum-free DMEM were added; this was incubated at 37 °C under 5% CO_2_. When 70% of the cells had developed CPEs, the liquid was discarded, the dish was washed three times with PBS, and the liquid was discarded. Then, 1 mL of 4% paraformaldehyde was added, the cells were fixed for 20 min, then washed three times with PBS. Next, the liquid was discarded, and 1 mL of 0.1% Triton X-100 was added. After 10 min, the dish was washed three times with PBS, and the liquid was discarded. Then, 1 mL of 5% bovine serum albumin (Einhausen, Germany) was added. The dish was covered for 30 min, washed three times with PBS, and the liquid was discarded. After that, 1 mL of PI-3 polyclonal antiserum (Pullman, WA, USA) diluted at 1:400 was added. This mixture was incubated at 37 °C for 1 h, the cells were washed three times with PBS, and the liquids were discarded. Next, an anti-fluorescence quenching agent (Biyuntian, Shanghai, China) was added dropwise, and observation was performed using a laser confocal microscope (Nikon C2+; Nikon, Tokyo, Japan).

### 2.5. Transmission Electron Microscopy

The virus-containing supernatants of the XJ21032-1 and XJ20055-3 isolates (20 μL) were obtained separately, and an appropriate amount was added dropwise to the copper grid of a Formav film and incubated for 5 min. The residual liquid was blotted with filter paper, negative staining with 2% phosphotungstic acid was performed, and the film was observed using an HT7800 transmission electron microscope.

### 2.6. Median Tissue Culture Infectious Dose Assay

The XJ21032-1 and XJ20055-3 virus liquids were diluted fold from 10^−1^ to 10^−10^, respectively. Using a second 96-well cell-culture plate, we inoculated approximately 8000 to 10,000 cells into each well and cultured them in an incubator at 37 °C under 5% CO_2_. When each well had reached confluence, the medium was discarded from the second plate, and the cells were washed three times with PBS. Next, we inoculated each well with 100 μL of the diluted virus solution from the corresponding well of the first 96-well cell-culture plate and adsorbed it for 2 h at 37 °C under 5% CO_2_. Then the virus solution was discarded, and the plate was washed three times with PBS. Next, 200 μL of DMEM were added to each well. After culturing at 37 °C under 5% CO_2_ for 5 to 6 days, the number of wells showing CPEs was determined using an inverted microscope. Finally, the results were calculated using the Reed–Muench method.

### 2.7. Next-Generation Sequencing and Sequence Analysis

The complete genome sequence for two isolates, XJ21032-1 and XJ20055-3, was determined by Shanghai Tanpu Biotechnology Co., Ltd. (Shanghai, China). The viral RNA was extracted and then fragmented into 300–500-bp nucleic acid fragments. Random reverse transcription was performed using the fragmented RNA. Sequencing junctions were attached to both ends of the transcribed cDNA fragments. Libraries were constructed, and bridge polymerase chain reaction amplification was performed, followed by Illumina Novaseq6000 PE150 sequencing. The sequences were sequenced using BBmap version 38.51 software for sequence comparison and removal of the corresponding rRNA, host, and bacterial sequences. The purified sequences were assembled using SPAdes version 3.14.1 and SOAPdenovo version 2.04 software. Finally, the assembled sequences were blast-annotated, and gene-annotated.

**Figure 4:**
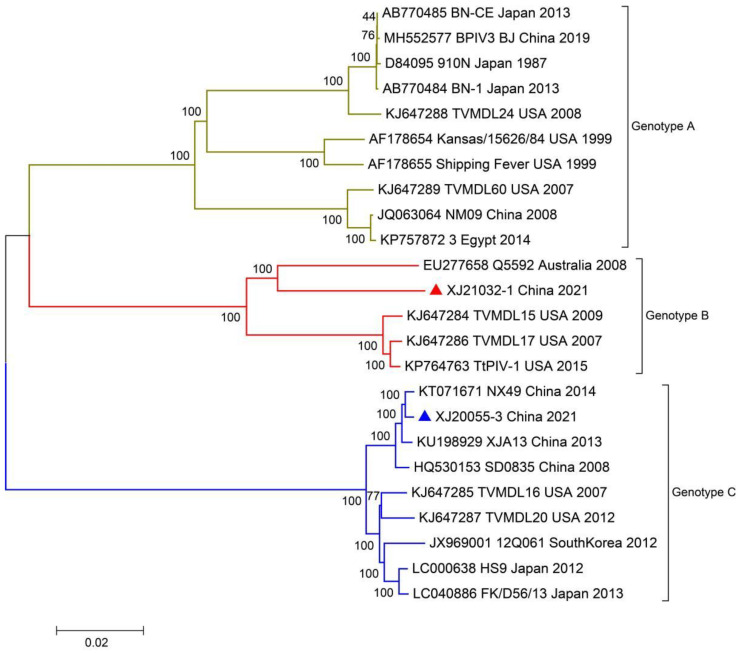
Phylogenetic analysis tree for the XJ21032-1 and XJ20055-3 isolates. In conjunction with other reference strains, the whole-genome nucleotide sequences of the XJ21032-1 and XJ20055-3 isolates were used to construct phylogenetic analysis trees using the NJ MEGA method 7.0 software. The lengths of the horizontal lines represent the genetic distance. The numbers at the nodes indicate the bootstrap percentage obtained after 1000 bootstrap replicates. Our isolates are marked with triangles.

Twenty-two whole-genome sequences of BPIV3 of genotypes A, B, and C were selected using GenBank (Table S1), and the genome sequences of the XJ21032-1 and XJ20055-3 isolates obtained during this study were compared for homology using the clustal W algorithm in MegAlign software. The neighbor-joining method was used to construct a phylogenetic analysis tree. The accession numbers of the reference sequence are shown in Figure 4.

## 3. Results

### 3.1. Virus Isolation and RT-PCR Identification

Twenty of the 407 nasal swabs were positive for matrix (M) gene detection by RT-PCR, whereas IBRV, BRSV, and BVDV were negative.

**Figure 1:**
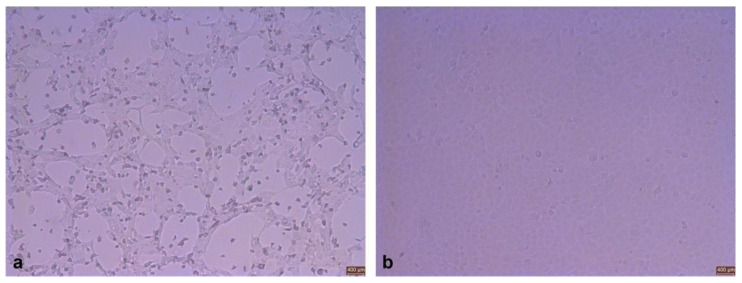
Observed cells after inoculation (×100). (**a**) Cytopathic effects. (**b**) Normal cells.

Nine of 20 BPIV3-positive samples had significant cytopathic lesions after blind passage for three generations, with rounded, crinkled, and stretched cells showing a reticular pattern; however, the control cells grew well (Figure 1).

M gene fragments of BPIV3 were detected from nine samples that produced cytopathic lesions, fragments that were consistent with the expected size of 686 bp band and matched the size of the target fragment. The polymerase chain reaction products were identified as *BPIV3* by a sequencing comparison and named XJ21032-1, XJ21032-18, XJ21032-19, XJ21032-20, XJ20055-3, XJ20055-6, XJ20046-7, XJ21031-9, and XJ20056-17. The sequencing results showed that XJ21032-1, XJ21032-18, XJ21032-19, and XJ21032-20 had high homology with the type B reference strain, and the isolates XJ20055-3, XJ20055-6, XJ20046-7, XJ21031-9, and XJ20056-17 had high homology with the type C reference strain.

### 3.2. Immunofluorescence Assay Identification

**Figure 2:**
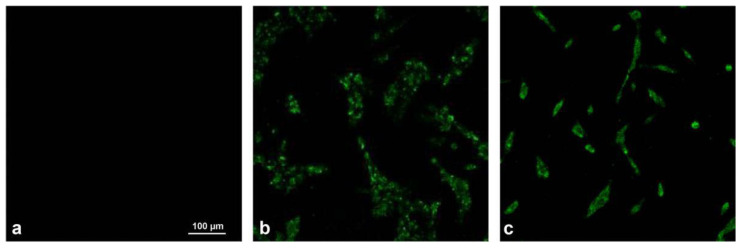
Viral immunofluorescence assay identification (×400). (**a**) Negative control. (**b**) XJ21032-1 isolate. (**c**) XJ20055-3 isolate.

When the cytopathic effect was observed in 70% of cells, BPIV3 polyclonal antiserum was incubated, followed by fluorescein isothiocyanate labeled secondary antibody (Solabao, Beijing, China). The specific green fluorescence was observed by laser confocal microscope. We confirmed that the virus could react with BPIV3-positive serum, thus proving it was BPIV3 (Figure 2).

### 3.3. Morphological Characterization of the Isolates

**Figure 3:**
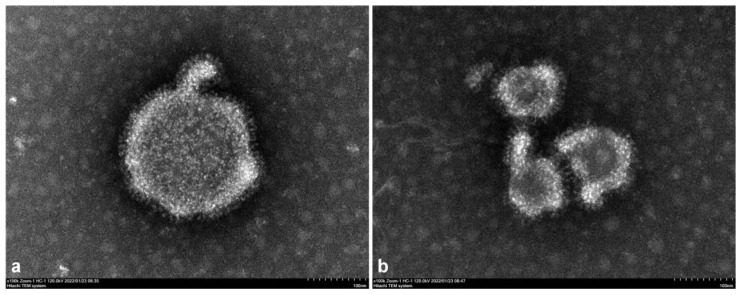
Transmission electron microscopy observation of the virus. (**a**) XJ21032-1 isolate. (**b**) XJ20055-3 isolate.

Transmission electron microscopy showed that the virus particles were approximately 150 nm in diameter and had a nearly spherical particle shape. A vesicular membrane tightly surrounded the nucleocapsid, and the morphology was similar to that of paramyxoviruses (Figure 3).

### 3.4. Median Tissue-Culture Infectious Doses

The median of virus tissue culture infection dose (TCID_50_) was determined in MDBK cells. We carried out a 10 times dilution of 10 dilution degrees, and 8 replicates were made for each dilution degree. The final virus titers of XJ21032-1 and XJ20055-3 were 10^7.9^ TCID50/mL, 10^8.4^ TCID_50_/mL, respectively, by the Reed–Muench method according to the CPE (Tables S2 and S3).

### 3.5. Sequence Analysis of Isolates

The Illumina sequencing results showed that the genomes of XJ21032-1 and XJ20055-3 had lengths of 15,512 bp and 15,479 bp, respectively, and GC contents of 44.89% and 42.13%, respectively. The average sequencing depths were 1725× and 317×, respectively. The sequences were submitted to GenBank with accession numbers ON081628 and OM632676, respectively. The genome was divided into six coding regions (N, P, M, F, HN, and L). In isolate XJ21032-1, the genes N, P, M, F, HN, and L had lengths of 1548 bp, 1791 bp, 1056 bp, 1623 bp, 1719 bp, and 6702 bp, respectively. In isolate XJ20055-3, these genes had lengths of 1548 bp, 1803 bp, 1056 bp, 1623 bp, 1719 bp, and 6774 bp, respectively.

The homology comparison revealed that XJ21032-1 had the highest nucleotide homology (93.4%) with the BPIV3 B-genotype reference strain Q5592 (EU277658) from Australia, 82.6% to 83.3% homology with the A-genotype reference strains (AB770485, MH552577, D84095, AB770484, KJ647288, AF178654, AF178655, KJ647289, JQ063064, KP757872), 92.1% to 93.4% homology with the B-genotype reference strains (EU277658, KP764763, KJ647284, KJ647286), and 80.9% to 81.2% homology with the C-genotype reference strains (KJ647285, KJ647287, JX969001, LC000638, LC040886, HQ530153, KU198929, KT071671). On the other hand, XJ20055-3 had the highest nucleotide homology (99.6%) with the BPIV3 C-genotype reference strain NX49 from Ningxia (KT071671), 82.1% to 82.4% homology with the A-genotype reference strains, 81.2% to 81.5% homology with the B-genotype reference strains, and 97.5% to 99.6% homology with the C-genotype reference strains.

Phylogenetic analysis showed that all isolates were divided into three genotypes, A, B, and C, and that each genotype could be further divided into subtypes. The A genotype was divided into three subtype branches; the A genotype BPIV3 BJ and NM09 isolates from China were in two different subtype branches. The B genotypes were divided into two subtype branches. The isolate XJ21032-1 from this study was in the same subtype branch as the Q5592 isolate from Australia, whereas the three B isolates from the United States were in the other subtype branch. The C genotypes were divided into two subtype branches, with the XJ20055-3 isolate being in the same subtype branch as the SD0835 isolate from Shandong, the XJA13 isolate from Xinjiang, and the NX49 isolate from Ningxia, which was found for the first time in China. The C isolates from the United States, Korea, and Japan were in the same subtype (Figure 4).

The N, F, and HN proteins were the major protective antigens of BPIV3. The XJ21032-1 isolate showed amino acid changes at 24 positions in the F protein, 20 in the HN protein, and 13 in the N protein compared with the highly homologous Q5592 reference strain. The XJ20055-3 isolate showed amino acid changes at one position in the F protein and three in the HN protein, but none in the N protein compared with the highly homologous NX49 reference strain (Table 2). The XJ20055-3 isolate had one amino acid change in the F protein, three amino acid changes in the HN protein, and none in the N protein compared with the highly homologous NX49 reference strain (Table 2).

## 4. Discussion

BPIV3 has been reported in the United States [7], Australia [9], Canada [5], and other developed countries with livestock industries and has caused significant economic losses. Because BPIV3 is very sensitive to the external environment, which makes it difficult to isolate in vitro, therefore, the number of BPIV3 isolates and amount of genomic data are limited worldwide. The nine strains of BPIV3 virus isolated in this study produced typical cytopathy on MDBK cells. After PCR identification and sequencing comparison, four strains had high homology with type B, and five had high homology with type C. One strain was randomly selected from the two groups of different genotypes. Morphological observation of the selected XJ21032-1 and XJ20055-3 isolates showed that they were similar to paramyxovirus. An indirect immunofluorescence test showed that they could react with BPIV3 polyclonal antiserum to produce specific green fluorescence. The TCID_50_ results indicated that they had good infectivity with MDBK cells.

The genomes of XJ21032-1 and XJ20055-3 were sequenced using next-generation (Illumina) sequencing technology and had genome lengths of 15,512 bp and 15,479 bp, respectively. Phylogenetic analysis revealed that isolate XJ21032-1 in this study was in the B-genotype branch with isolate Q5592 from Australia and isolates TVMDL15, TVMDL17, and TtPIV-1 from the United States. Isolate XJ21032-1 was in the same B-genotype branch as the Q5592 isolate from Australia, whereas the three B isolates from the United States belonged to another branch. Isolates TVMDL15, TVMDL17, and TtPIV-1 from the United States and isolate XJ21032-1 were genetically related to the isolate from Australia in the same subtype branch; however, the three B isolates from the United States were in another branch. The XJ20055-3 isolate of this study isolates TVMDL16 and TVMDL20 from the United States, 12Q061 from Korea, HS9 and FK/D56/13 from Japan, isolates SD0835 and XJA13 from China, and isolate NX49 were in the same C-genotype branch. Among these, the XJ20055-3 isolate was the same as the Chinese C genotype. The genetic relationship of the isolates in a given subtype branch was relatively close. In contrast, the C-type isolates from the United States, Korea, and Japan were in different branches. This pattern may be due to geographical factors. A comparison of homologies revealed that the highest homology of the XJ21032-1 isolate (93.4%) was with the Q5592 reference strain from Australia, and the highest homology of isolate XJ20055-3 (99.6%) was with the NX49 reference strain from Ningxia, China.

When genotypes B and C were first discovered, they were thought to be the result of geographical isolation and endemic only to fixed regions [10]; however, an increasing number of epidemiological investigations have shown that genotypes B and C are present in multiple countries. During this study, a BPIV3 B-genotype strain was successfully isolated and sequenced, thus enriching our knowledge of BPIV3 genotypes in China. Furthermore, three genotypes of BPIV3 have been found in the United States [16] and Argentina [15]. In addition to geographical isolation, international animal trade may be responsible for the worldwide spread of different BPIV3 genotypes; therefore, more genome sequences of BPIV3 isolates with different genotypes from different regions must be obtained for analysis. During this study, the isolated XJ21032-1 strain was traced, and we learned that the cattle farm where the isolate originated was raising cattle from both Australia and the local area. This background is consistent with our phylogenetic analysis of this isolate, which places it in the same branch as the reference strain Q5592 from Australia; however, it is worth considering that the homology between this B-type isolate and the B-type isolates from the United States and Australia is low (92.1–93.4%). In contrast, the homology among the three B-type isolates from the United States is greater than 99%. Although genotype B had not been reported in China before this study, and that it has high homology with the Australian isolate, we still cannot conclude that the source of the B-type isolate based on this information alone. We still require additional clinical data on the domestic B-type isolates to determine its source. The isolated XJ20055-3 strain was in the same branch of genetic evolution as the C-type isolate from China; therefore, it is likely that the strain evolved from the C-type strain from China and that some of the changes in its gene sequence may be attributable to the different geographical environments of the regions of origin. 

No studies have elucidated whether differences in virulence exist among the BPIV3 A, B, and C genotypes; however, in the United States [16] and Argentina [15], differences between genotypes in neutralization potency have been found by virus neutralization tests, suggesting that there may be differences in antigenicity between the genotypes. There is no BPIV3 vaccine in China, but the BPIV3 vaccine made from the A strain has long been used in clinical practice in the United States. Although the vaccine is effective in clinical practice, Newcomer et al. [7] found that the prevalence of genotypes B and C of BPIV3 in herds in the United States is increasing because of the use of only genotype A in the BPIV3 vaccine. Studies of BPIV3 isolates performed in several provinces in China found that BPIV3 was predominantly of the genotype C, with some genotype A; however, the present study confirmed the presence of genotype B, suggesting that genotype subtypes should be determined when developing BPIV3 vaccines so that the developed vaccines are effective against all genotypes. Because the N, F, and HN proteins are the main protective antigens of the BPIV3 virus and are associated with the ability of the virus to replicate, adsorb, and penetrate, it is extremely important to focus attention on the amino acid changes occurring in these proteins [20,21]. In this study, the amino acid sequences of the N, F, and HN proteins of the XJ21032-1 and XJ20055-3 isolates were compared, and several amino acid changes were found. Further studies are needed to reveal these amino acid changes’ effects on the virus.

In this study, nine BPIV3 strains were isolated, and the XJ21032-1 and XJ20055-3 isolates were selected for genome sequencing. They were genotypes B and C, respectively, by means of a phylogenetic analysis, which proved the existence of the BPIV3 B genotype in China. This is the first report of BPIV3 B-genotype strains in China. These results may help trace the origin of BPIV3 and understand the differences between genotypes, thus creating a foundation for vaccine development.

## Figures and Tables

**Table 1:** Primer sequences.

Gene	Primer	Sequence (5′-3′)	Product Size (bp)	Annealing Temperature (°C)
IBRV-gB	F1	F:CACGGACCTGGTGGACAAGAAG	656	60
	R1	R:CTTCGATCACGCAGTCGCTCA		
BVDV-5’ UTR	F1	F:AGCCATGCCCTTAGTAGGACT	290	55
	R1	R:ACTCCATGTGCCATGTACA		
BRSV-F	F1	F:AATCRACATGCAGTGCAGTTAG	711	50
	R1	R:TTTGGTCATTYGTTATAGGCA		
BPIV3-M	F1	F:TTAGAYATAGAAGTRAGAAGAAC	686	50
	R1	R:ATRTTTGGGTARTATCTRAAYTCAC		

**Table 2:** Amino acid differences between F, HN, and N proteins.

Protein Name	XJ21032-1 and Q5592 (Position)	XJ20055-3 and NX49 (Position)
F	I(5)V; V(8)M; A(13)T; M(17)L; R(24)K; L(62)S; E(91)D; I(92)V; R(222)G; I(226)V; V(328)I; I(352)M; D(442)N; L(465)S; Y(488)H; A(492)V; V(509)A; V(513)M; K(517)R; A(521)D; Q(522)R; P(525)H; S(526)L; K(527)N	T(492)I
HN	N(12)S; G(21)R; H(24)Y; I(28)A; T(29)A; V(35)A; F(36)L; T(38)A; I(39)T; I(42)L; L(67)Q; E(74)T; Q(81)R; V(89)A; R(131)K; N(250)D; N(385)D; M(382)K; I(436)L; L(487)S	N(216)D; K(254)R; N(374)D
N	A(150)T; S(151)A; G(207)S; G(401)S; N(402)S; A(424)V; T(454)N; G(455)S; S(460)P; A(461)T; T(464)I; N(469)T; P(491)T	None

IUPAC code: A = alanine; B = aspartic acid; C = cysteine; D = aspartic acid; E = glutamic acid; F = phenylalanine; G = glycine; H = histidine; I = isoleucine; K = lysine; L = leucine; M = methionine; N = asparagine; P = proline; Q = glutamine; R = arginine; S = serine; T = threonine; V = valine; W = tryptophan.

## Data Availability

The sequences generated in this study have been submitted to GenBank (ncbi.nlm.nih.gov) under accession numbers ON081628 and OM632676. Information on the sequences used in the analysis of this study is provided in Table S1.

## Supplementary Materials

The following supporting information can be downloaded at: https://www.mdpi.com/article/10.3390/v14102221/s1, Table S1: Information on sequences used in this study; Table S2: The median tissue culture infectious dose (TCID50) of the XJ21032-1 isolate; Table S3: The TCID50 of the XJ20055-3 isolate.
