# Supplementary material for: Isolation, Identification, and Genetic Phylogenetic Analysis of Two Different Genotypes of Bovine Parainfluenza 3 Virus in China
Source: Viruses. 2022 Oct 9;14(10):2221. doi: 10.3390/v14102221 (PMC9607367; doi:10.3390/v14102221)
Supplement: Supplementary file 1 [file viruses-14-02221-s001.zip › viruses-1888647-supplementary.pdf]

Supplementary Information

The sequences generated in this study have been submitted to GenBank under accession numbers ON081628 and OM632676. The information about the sequences used in the analysis of this study is shown in Table S1.

Table S1 Information on sequences used in this study

Virus	GenBank accession number	Country	Year isolation	Genotype	Reference
BN-CE	AB770485	Japan	2013	A	Ohkura,T et al.2013
BPIV3 BJ	MH552577	China	2019	A	Liu,C et al.2019
910N	D84095	Japan	1987	A	Sakai,Y et al.1987
BN-1	AB770484	Japan	2013	A	Ohkura,T et al.2013
TVMDL24	KJ647288	USA	2008	A	Neill,J.D et al.2015
Kansas/15626/84	AF178654	USA	1999	A	Bailly,J.E et al.2000
Shipping Fever	AF178655	USA	1999	A	Bailly,J.E et al.2000
TVMDL60	KJ647289	USA	2007	A	Neill,J.D et al.2015
NM09	JQ063064	China	2008	A	Wen,Y.J et al.2012
3 Egypt 2014	KP757872	Egypt	2014	A	Sobhy,N.M et al.2014
Q5592	EU277658	Australia	2008	B	Horwood,P.F et al.2008
XJ21032-1	ON081628	China	2021	B	This study
TVMDL15	KJ647284	USA	2009	B	Neill,J.D et al.2015
TVMDL17	KJ647286	USA	2007	B	Neill,J.D et al.2015
TtPIV-1	KP764763	USA	2015	B	Eberle,K.C et al.2015
NX49	KT071671	China	2014	C	Wen,X.B et al.2015
XJ20055-3	OM632676	China	2021	C	This study
XJA13	KU198929	China	2013	C	Sun,M et al.2015
SD0835	HQ530153	China	2008	C	Zhu,Y.M et al.2011
TVMDL16	KJ647285	USA	2007	C	Neill,J.D et al.2015
TVMDL20	KJ647287	USA	2012	C	Neill,J.D et al.2015
12Q061	JX969001	SouthKorea	2012	C	Oem,J.K et al.201
HS9	LC000638	Japan	2012	C	Konishi,M et al.2014
FK/D56/13	LC040886	Japan	2013	C	Konishi,M et al.2014

Table S2. The median tissue culture infectious dose ($TCID_{50}$) of the XJ21032-1 isolate

Viral dilution	Cell pores, no.	No CPE holes, no.	CPE holes, no.	Cumulative CPE-free holes, no.	Cumulative CPE holes, no.	Cumulative cell pores, no.	Cumulative CPE, %
10^{-1}	8	0	8	0	52	52	100.00
10^{-2}	8	0	8	0	44	44	100.00
10^{-3}	8	0	8	0	36	36	100.00
10^{-4}	8	0	8	0	28	28	100.00
10^{-5}	8	0	8	0	20	20	100.00
10^{-6}	8	0	8	0	12	12	100.00
10^{-7}	8	5	3	5	4	9	44.44
10^{-8}	8	7	1	12	1	13	7.69
10^{-9}	8	8	0	20	0	20	0.00
10^{-10}	8	8	0	28	0	28	0.00

Table S3. The $TCID_{50}$ of the XJ20055-3 isolate

Viral dilution	Cell pores, no.	No CPE holes, no.	CPE holes, no.	Cumulative CPE-free holes, no.	Cumulative CPE holes, no.	Cumulative cell pores, no.	Cumulative CPE %
10^{-1}	8	0	8	0	55	55	100.00
10^{-2}	8	0	8	0	47	47	100.00
10^{-3}	8	0	8	0	39	39	100.00
10^{-4}	8	0	8	0	31	31	100.00
10^{-5}	8	0	8	0	23	23	100.00
10^{-6}	8	0	8	0	15	15	100.00
10^{-7}	8	3	5	3	7	10	70.00
10^{-8}	8	6	2	9	2	11	18.18
10^{-9}	8	8	0	17	0	17	0.00
10^{-10}	8	8	0	25	0	25	0.00
